# Ein Case-Management-Fragebogen für Angehörige geriatrischer Patienten

**DOI:** 10.1007/s00391-021-01871-1

**Published:** 2021-03-18

**Authors:** Julian Schmitt, Nicole Warkentin, Denise Wilfling, Jost Steinhäuser, Katja Götz

**Affiliations:** Institut für Allgemeinmedizin, Universitätsklinikum Schleswig-Holstein, Campus Lübeck, Ratzeburger Allee 160, Haus 50, 23538 Lübeck, Deutschland

**Keywords:** Fragebogenentwicklung, Care-und-Case-Management, Pflegende Angehörige, Psychometrik, Geriatrische Patienten, Questionnaire development, Care and case management, Family caregivers, Psychometrics, Geriatric patients

## Abstract

**Hintergrund:**

Versorgungskonzepte, die zu einer Entlastung der pflegenden Angehörige beitragen, werden dringend benötigt. „**R**egional **u**nunterbrochen **b**etreut **i**m **N**etz“ (RubiN), welches mit einem Care-und-Case-Management in Ärztenetzen die Versorgung von geriatrischen Patienten unterstützen soll, zielt auch darauf ab, Angehörige zu entlasten. Ziele waren daher die Entwicklung und psychometrische Überprüfung eines Fragebogens, der die Zufriedenheit und Akzeptanz mit der Versorgung durch ein Case Management (CM) aus Perspektive der Angehörigen erfasst.

**Methodik:**

Es wurde ein „Mixed-methods“-Design zur Konzeptualisierung des Fragebogens gewählt. Neben der Entwicklung des Fragebogens anhand qualitativer Interviews sowie eigener Projekt- und Studienerfahrungen erfolgten die Pilotierung und anschließende psychometrische Überprüfung des Fragebogens in den 5 teilnehmenden RubiN-Ärztenetzen. Von Mai bis August 2020 fand die Befragung in den Ärztenetzen statt. Jedes der 5 beteiligten Ärztenetze erhielt ein Set mit je 50 Fragebogen für Angehörige. Das Fragebogenkonstrukt wurde psychometrisch überprüft.

**Ergebnisse:**

Der konzipierte Fragebogen bestand aus 11 Items. Insgesamt nahmen 137 Angehörige an der Befragung teil (Rücklaufquote 55 %). Die Angehörigen waren mit der angebotenen Versorgung sehr zufrieden (78,1 %). Des Weiteren zeigten die Daten sehr geringe fehlende Werte auf. Die 11 Items des Fragebogens luden auf 2 Faktoren. Faktor 1 „Auswirkungen durch die Koordination“ wies eine interne Konsistenz von 0,843 und Faktor 2 „Erreichbarkeit“ eine interne Konsistenz von 0,683 auf.

**Diskussion:**

Der Fragenbogen umfasst die Akzeptanz und Zufriedenheit der angebotenen Versorgungselemente für Angehörige mit einem geriatrischen CM und zeichnet sich mit 11 Items durch seine Kürze aus.

## Abstract (en)

**Background:**

To ease the burden for family caregivers healthcare concepts are urgently needed. The healthcare concept continuous care in a regional network (RubiN) offers a care and case management in physician networks for geriatric patients and aims to relieve family caregivers. The aim of the study was to develop and to test the psychometric properties of a questionnaire that evaluates the satisfaction and acceptance of the healthcare provided by case management (CM) from the perspective of family caregivers.

**Methods:**

A mixed methods design was used to develop the questionnaire based on qualitative interviews, a literature search and own project experiences. After a pilot run the subsequent data acquisition took place in the participating RubiN physician networks from May to August 2020. Each of the 5 participating networks received a set of 50 questionnaires for family caregivers. The psychometric properties of the questionnaire were evaluated.

**Results:**

The questionnaire consisted of 11 items. A total of 137 family caregivers participated in the survey (response rate 55%). Overall, the family caregivers were very satisfied (78.1%) with the care offered. There were only a few missing values in the data. The 11 items in the questionnaire loaded on 2 factors. The first factor called effects from coordination showed an internal consistency of 0.843 and for the second factor called accessibility an internal consistency of 0.683 was observed.

**Conclusion:**

The questionnaire with 11 items is characterized by its brevity and includes the acceptance and satisfaction of the care elements offered for family caregivers in a geriatric CM.

## „Hinführung zum Thema“

Die Versorgungsbedarfe von geriatrischen Patienten sind komplex. Angehörige übernehmen dabei häufig die Langzeitbetreuung ihres zu pflegenden Angehörigen. Ein Care Management kann dabei unterstützen, Angehörige zu entlasten. Regional ununterbrochen betreut im Netz (RubiN), gefördert vom Innovationsfonds, untersucht u. a., inwiefern ein Care Management eine Entlastung darstellen, und inwiefern die Angehörigen mit dieser Art von Versorgung zufrieden sind. In diesem Beitrag wird ein dazu entwickelter Fragebogen vorgesellt.

## Hintergrund

Angehörige übernehmen häufig die Langzeitbetreuung ihrer ihnen nahestehenden Personen, um eine möglichst lange Versorgung in der eigenen Häuslichkeit zu gewährleisten. Untersuchungen im Hinblick auf die Wohnwünsche im Alter bestätigen, dass die Mehrheit der Befragten anstrebt, so lange wie möglich in ihrer häuslichen Umgebung zu wohnen [12]. Laut der nationalen Versichertenbefragung der Kassenärztlichen Bundesvereinigung 2018 befanden sich 18 % der 1883 Befragten in der Rolle des pflegenden Angehörigen [10]. Angehörige, die in die Pflege nahestehender Personen eingebunden sind, stellen das Rückgrat der Versorgung Pflegebedürftiger im ambulanten Setting dar.

Daher bergen die von den pflegenden Angehörigen wahrgenommenen Belastungsfaktoren, die sich multidimensional präsentieren können, schwerwiegende Konsequenzen für die häusliche Versorgungssituation [2]. Unter anderem zeigt sich für die pflegenden Angehörigen ein hohes Risiko, sowohl physisch als auch psychisch zu erkranken [6]. Daher werden Versorgungskonzepte, die zu einer Entlastung der pflegenden Angehörige beitragen, dringlich benötigt.

Eine Möglichkeit der Entlastung kann durch ein Care-und-Case-Management entstehen, welches sich auch an die Angehörigen als Zielgruppe richtet. In Anlehnung an die „Deutsche Gesellschaft für Care- und Case-Management e. V.“ wird unter Care Management ein systembezogenes Vorgehen und unter Case Management (CM) ein fallbezogenes Vorgehen verstanden [7]. In verschiedenen internationalen Übersichtsarbeiten konnten die positiven Effekte durch CM auf Angehörige gezeigt werden, die einerseits die Stärkung der eigenen Gesundheit und andererseits die Reduzierung von Belastungsfaktoren betreffen [3, 4, 14].

In dem Projekt „**R**egional **u**nunterbrochen **b**etreut **i**m **N**etz“ (RubiN), welches darauf abzielt, mit einem Care-und-Case-Management die Versorgung von hausärztlich betreuten, geriatrischen Patienten in einem Ärztenetz zu strukturieren, zu koordinieren, Hilfsangebote aufzuzeigen und zu beantragen, spielt auch die Angehörigenperspektive eine zentrale Rolle [13]. Detaillierte Informationen zum Projekt können bei Laag et al. nachgelesen werden [13].

Standardisierte Instrumente, die aus Perspektive der Angehörigen die Umsetzung solcher Versorgungsmodelle insbesondere des CM im Versorgungsalltag und die daraus resultierenden Konsequenzen für die Angehörigen messen, werden dringend benötigt. Ziele der vorliegenden Studie waren daher die Entwicklung und psychometrische Überprüfung eines Fragebogens, der die Zufriedenheit und Akzeptanz mit der Versorgung durch ein CM aus Perspektive der Angehörigen erfasst, um die Ergebnisqualität zu messen. Folgende Forschungsfragen – unterteilt nach qualitativ und quantitativ – kamen dabei zum Einsatz:

- Qualitative Forschungsfrage: Welche Themenkomplexe müssen bei der Konzeptualisierung eines Fragebogens zur Erfassung der Zufriedenheit und Akzeptanz durch die Versorgung mit einem CM Berücksichtigung finden?
- Quantitative Forschungsfrage: Inwiefern lässt sich der Fragebogen zur Erfassung der Zufriedenheit und Akzeptanz durch die Versorgung mit einem CM bei der Zielgruppe der Angehörigen operationalisieren?

## Methodik

Die vorliegende Studie war als „Mixed-methods“-Design konzipiert. Die Zielgruppe der vorliegenden Befragung waren Angehörige von hausärztlich betreuten, geriatrischen Patienten, die in das Projekt RubiN eingeschrieben waren und eine Versorgung durch ein CM erhielten.

### Fragebogenentwicklung

Für die Entwicklung des Fragebogens wurden persönliche Interviews mit Care-und-Case-Managern (CCM) und Patienten mittels eines halbstandardisierten Leitfadens durchgeführt. Die Fragen waren identisch und zielten darauf ab herauszufinden, welche Entlastung ein CM für Angehörige bringt, und welche Angebote Angehörigen durch die CCM unterbreitet werden. Der Interviewleitfaden wurde auf Basis des existierenden Musterfachcurriculum Geriatrie zur GeriNurse^1^, in dem die Aufgaben beschrieben sind, sowie aus eigenen Studienerfahrungen mit einem anderen innovativen Versorgungsmodell hinsichtlich der Einbindung von Angehörigen entwickelt [19]. Der wissenschaftstheoretische Zugang zu den Interviews lag auf dem interpretativen Paradigma. Die Interviews dienten als Vorstudie für die inhaltliche Ausgestaltung des Fragebogens. Die Rekrutierung der CCM und der Patienten erfolgte im GeriNet e. V. in Leipzig. Sie wurden im Vorfeld über die Studie und datenschutzrechtliche Aspekte aufgeklärt. Dieses Netz bietet seit Jahren eine Versorgung für geriatrische Patienten an, die durch ein CCM begleitet wird. Eine schriftliche Zustimmung war Voraussetzung für die Teilnahme. Die Interviews fanden im November 2019 in Leipzig statt. Alle Interviews wurden von demselben Interviewer (KG) durchgeführt. Die Gespräche wurden digital aufgezeichnet und pseudonymisiert als Volltext orthographisch transkribiert. Die Auswertung der Interviews erfolgte mittels qualitativer Inhaltsanalyse nach Mayring [15]. Zunächst wurde deduktiv anhand des Leitfadens ein Kategoriensystem erstellt, welches während des Auswertungsprozesses durch neue Kategorien (induktiv) ergänzt wurde. Die Transkripte wurden unabhängig von 2 Personen (KG (Soziologin und Versorgungsforscherin), JS (Student der Humanmedizin)) codiert und in 2 Konsensrunden diskutiert, sodass am Ende der Auswertung die inhaltliche Ausgestaltung der Themenkomplexe feststand. Im Anschluss daran wurde aus den Themenkomplexen Aussagen für den Fragebogen entwickelt.

Der Fragebogen beinhaltete 11 Aussagen zu Akzeptanz und Zufriedenheit mit der Versorgung durch das CM. Die Antwortmöglichkeiten zu den verschiedenen Items wurden mit einer 6‑stufigen Likert-Skala (1 ≙ trifft voll und ganz zu bis 6 ≙ trifft gar nicht zu) gemessen. Ergänzt wurde der Fragebogen mit Fragen zu Alter, Geschlecht und verwandtschaftlichem Grad zu den geriatrischen Patienten.

### Pilotierung des Fragebogens

Im Januar 2020 erfolgte mittels kognitiver Interviews im Sinne des „concurrent think aloud“ die Überprüfung der Sinnhaftigkeit und Praktikabilität einzelner Fragen [11, 16]. Hierzu wurden CCM sowie Angehörige telefonisch gebeten, an der Pilotierung teilzunehmen, um bei Bedarf die Adaptierung einzelner Items vorzunehmen.

### Psychometrische Eigenschaften des finalen Fragebogens

Bei der anschließenden Befragung wurden neben der Durchführung einer Itemanalyse die psychometrischen Eigenschaften des Fragebogens hinsichtlich der faktoriellen Validität (Faktorenanalyse) und Reliabilität bestimmt.

### Rekrutierung

Mit dem pilotierten Fragebogen wurde von Mai bis August 2020 eine quantitative Befragung durchgeführt. Die Rekrutierung erfolgte über die 5 teilnehmenden Ärztenetze in RubiN. Die Projektkoordinatoren und Geschäftsführer dieser Netze wurden im Vorfeld gebeten, die Rekrutierung zu unterstützen. Jedes der 5 Netze erhielt aufgrund der Vergleichbarkeit hinsichtlich der zu verteilenden Anzahl ein Set mit je 50 Fragebogen für Angehörige. Konsekutiv wurden die Fragebogen durch die CCM an die Angehörigen bei Hausbesuchen verteilt. Die Angehörigen erhielten neben dem Fragebogen einen vorfrankierten Rückumschlag, mit dem der Fragebogen an die Studienzentrale zurückgesendet werden konnte.

### Statistische Analyse

Die Dateneingabe erfolgte durch JS, und die Auswertung wurde von KG durchgeführt. Für die Analysen wurde das Statistikprogramm SPSS 26.0 (SPSS Inc., IBM) verwendet. Im ersten Schritt erfolgte eine deskriptive Auswertung der soziodemografischen Variablen; für stetige Variablen wurden der Mittelwert und die Standardabweichung und für kategoriale Daten wurden die Häufigkeit und der prozentuale Anteil angegeben. Für die einzelnen Items des Fragebogens wurden die Itemkennwerte ermittelt. Neben der Verteilung der Antworthäufigkeiten wurden der Mittelwert, die Standardabweichung sowie die Kurtosis und Schiefe berechnet. Eine explorative Faktorenanalyse mit Hauptkomponentenextraktion wurde durchgeführt, um die Dimensionalität der Items zu prüfen. Die Zahl der Komponenten wurde durch den Eigenwert >1 determiniert. Die Komponenten wurden einer Varimax-Rotation unterzogen. Des Weiteren wurden das Kaiser-Meyer-Olkin-Kriterium (KMO) zur Stichprobeneignung und der Bartlett-Test auf Sphärizität geprüft [1]. Alle Items, die eine Faktorenladung >0,5 aufwiesen, wurden dem jeweiligen Faktor zugeordnet. Die interne Konsistenz wurde durch das Cronbachs α ermittelt [5]. Dabei stehen Werte >0,8 für eine gute, >0,6 für eine akzeptable und >0,4 für eine schlechte interne Konsistenz. Fehlende Werte bis 10 % galten als vernachlässigbar.

### Ethische Aspekte

Die vorliegende Studie wurde von der Ethikkommission der Universität zu Lübeck positiv begutachtet (Antragsnummer: 19-282).

## Ergebnisse

### Fragebogenentwicklung

Bei der Entwicklung des Fragebogens wurden 7 Interviews mit CCM und 2 mit Patientinnen durchgeführt. Die CCM waren im Schnitt 36 Jahre alt, 2 männlichen und 5 weiblichen Geschlechts. Die teilnehmenden Patientinnen waren ausschließlich weiblich und im Durchschnitt 82 Jahre alt. Die Interviews dauerten zwischen 20 und 120 min. Insgesamt wurden verschiedene Themen geäußert, die für einen Fragebogen, der die Angehörigen berücksichtigt, relevant sind. Dies waren Sinnhaftigkeit, Akzeptanz und Umsetzbarkeit der Angebote, Erreichbarkeit des CCM, Entlastung durch das CM sowie allgemeine Zufriedenheit. Die verschiedenen Themen mündeten in verschiedene Aussagen, z. B.

- Ich werde durch den Versorgungskoordinator entlastet.
- Ich weiß, wie ich den Versorgungskoordinator erreichen kann.
- Die vom Versorgungskoordinator vorgeschlagenen Angebote waren gut umsetzbar.

Die detaillierten Formulierungen sind hierzu in Tab. 1 zu finden.

**Table 1:** 

Items zur Zufriedenheit und Akzeptanz	1 *n* (%)	2 *n* (%)	3 *n* (%)	4 *n* (%)	5 *n* (%)	6 *n* (%)	Fehlend *n* (%)	MW (SD)	Schiefe	Kurtosis
Ich werde durch den Versorgungskoordinator entlastet	70 (51,1)	37 (27,0)	16 (11,7)	8 (5,8)	2 (1,5)	2 (1,5)	2 (1,5)	1,82 (±1,11)	1,57	2,45
Ich habe das Gefühl, dass sich die Situation meines/meiner Mannes/Frau/meines Partners durch den Versorgungskoordinator verbessert hat	56 (40,9)	40 (29,2)	25 (18,2)	7 (5,1)	2 (1,5)	3 (2,2)	4 (2,9)	2,01 (±1,15)	1,35	1,96
Die Empfehlungen des Versorgungskoordinators sind sinnvoll	94 (68,6)	36 (26,3)	5 (3,6)	–	–	–	2 (1,5)	1,34 (±0,55)	1,35	0,89
Ich würde den Versorgungskoordinator weiterempfehlen	120 (87,6)	13 (9,5)	4 (2,9)	–	–	–	–	1,15 (±0,44)	2,95	8,32
Ich weiß, wie ich den Versorgungskoordinator erreichen kann	122 (89,1)	14 (10,2)	1 (0,7)	–	–	–	–	1,12 (±0,34)	2,95	8,47
Die vom Versorgungskoordinator angebotenen Dienste/Leistungen sind sinnvoll für meine(n) Mann/Frau/Partner	89 (65,0)	37 (27,0)	5 (3,6)	2 (1,5)	–	–	4 (2,9)	1,40 (±0,64)	1,72	3,19
Die vom Versorgungskoordinator vorgeschlagenen Angebote waren gut umsetzbar	78 (56,9)	42 (30,7)	10 (7,3)	2 (1,5)	1 (0,7)	1 (0,7)	3 (2,2)	1,57 (±0,85)	2,13	6,47
Die Erreichbarkeit des Versorgungskoordinators war gut	109 (79,6)	26 (19,0)	1 (0,7)	–	–	–	1 (0,7)	1,21 (±0,42)	1,76	1,95
Das durch den Versorgungskoordinator ausgehändigte Infomaterial war verständlich	92 (67,2)	42 (30,7)	1 (0,7)	–	–	–	2 (1,5)	1,33 (±0,49)	0,95	−0,61
Die Besuchstermine des Versorgungskoordinators sind zu einer angemessenen Uhrzeit	106 (77,4)	24 (17,5)	4 (2,9)	–	–	–	3 (2,2)	1,24 (±0,49)	1,97	3,16
Insgesamt bin ich mit der Betreuung durch den Versorgungskoordinator zufrieden	107 (78,1)	27 (19,7)	3 (2,2)	–	–	–	–	1,24 (±0,48)	1,82	2,54

*1* trifft voll und ganz zu bis *6* trifft gar nicht zu, *n* Anzahl, *MW* Mittelwert, *SD* Standardabweichung

### Pilotierung

Es wurden kognitive Interviews (3 CCM und 2 Angehörige) geführt. Der Fragebogen wurde insgesamt als umfassend für die Abbildung der Angehörigenperspektive wahrgenommen. Die Denomination CCM wurde durch die Bezeichnung „Versorgungskoordinator“ bzw. in 2 Netzen durch „Gesundheitshelfer“ ausgetauscht, da dies als gängige Bezeichnung in den einzelnen Netzen genutzt wurde. Ansonsten erfolgte keine inhaltliche Veränderung durch die Pilotierung.

### Itemanalyse, Reliabilität und Faktorenstruktur des finalen Fragebogens

An der Befragung nahmen 137 Angehörige teil. Dies entsprach einer Rücklaufquote von 55 %. Von den 137 Angehörigen waren 100 (73,0 %) weiblich und 36 (26,3 %) männlich. Das durchschnittliche Alter betrug 66,0 Jahre. Als Grad der Verwandtschaft wurde am häufigsten Tochter/Sohn (54,7 %) oder Ehepartner (33,6 %) angegeben. Detaillierte soziodemografische Angaben sind Tab. 2 zu entnehmen.

**Table 2:** 

Variablen		Anzahl (%)	MW (SD)
Geschlecht	Männlich	36 (26,3)	–
	Weiblich	100 (73,0)	–
	Fehlend	1 (0,7)	–
Alter (Jahre)	–	–	66,0 (±13,6)
	Min–Max	–	25–90
	Fehlend	3 (2,2)	–
Grad der Verwandtschaft	Tochter/Sohn	75 (54,7)	–
	Ehepartner	46 (33,6)	–
	Lebensgefährte	5 (3,6)	–
	Enkel	2 (1,5)	–
	Nichte/Neffe	1 (0,7)	–
	Fehlend	8 (5,8)	–

*n* Anzahl, *MW* Mittelwert, *SD* Standardabweichung

Der Fragebogen zur Erfassung der Akzeptanz und Zufriedenheit durch die Versorgungskoordinatoren/Gesundheitshelfer beinhaltete 11 Items und ist detailliert in Tab. 2 dargestellt. Die Mittelwerte für die einzelnen Antwortmöglichkeiten lagen zwischen 1,12 („Ich weiß, wie ich den Versorgungskoordinator erreichen kann“) und 2,01 („Ich habe das Gefühl, dass sich die Situation meines/meines Mannes/Frau/Partner durch den Versorgungskoordinator verbessert hat.“).

Die explorative Faktorenanalyse, dargestellt in Tab. 3, ergab eine Zweifaktorenlösung mit einer erklärten Gesamtvarianz R^2^ von 59,00 % (KMO = 0,828; Bartlett-Test auf Sphärizität *p* < 0,001). Für Faktor 1 „Auswirkungen durch die Koordination“ lagen die Ladungen zwischen 0,703 („Insgesamt bin ich mit der Betreuung durch den Versorgungskoordinator zufrieden.“) und 0,786 („Ich werde durch den Versorgungskoordinator entlastet.“) mit einer internen Konsistenz von 0,843. Bei Faktor 2 „Erreichbarkeit“ ergaben sich Faktorenladungen zwischen 0,624 („Das durch den Versorgungskoordinator ausgehändigte Infomaterial war verständlich.“) und 0,755 („Die Erreichbarkeit des Versorgungskoordinators war gut.“) mit einer internen Konsistenz von 0,683.

**Table 3:** 

Items zu Zufriedenheit und Akzeptanz	KMO	Faktorladungen	
		F1	F2
Ich werde durch den Versorgungskoordinator entlastet	0,834	*0,786*	−0,067
Ich habe das Gefühl, dass sich die Situation meines/meiner Mannes/Frau/meines Partners durch den Versorgungskoordinator verbessert hat	0,776	*0,782*	−0,108
Die Empfehlungen des Versorgungskoordinators sind sinnvoll	0,863	*0,721*	0,363
Ich würde den Versorgungskoordinator weiterempfehlen	0,847	*0,720*	0,283
Ich weiß, wie ich den Versorgungskoordinator erreichen kann	0,636	−0,029	*0,728*
Die vom Versorgungskoordinator angebotenen Dienste/Leistungen sind sinnvoll für meine(n) Mann/Frau/Partner	0,866	*0,737*	0,341
Die vom Versorgungskoordinator vorgeschlagenen Angebote waren gut umsetzbar	0,918	*0,717*	0,222
Die Erreichbarkeit des Versorgungskoordinators war gut	0,777	0,184	*0,755*
Das durch den Versorgungskoordinator ausgehändigte Infomaterial war verständlich	0,793	0,442	*0,624*
Die Besuchstermine des Versorgungskoordinators sind zu einer angemessenen Uhrzeit	0,754	0,132	*0,647*
Insgesamt bin ich mit der Betreuung durch den Versorgungskoordinator zufrieden	0,849	*0,703*	0,367

*KMO* Kaiser-Meyer-Olkin-Kriterium, *F1* Auswirkungen durch die Koordination, *F2* Erreichbarkeit

## Diskussion

Die Entwicklung und Bestimmung der psychometrischen Eigenschaften zu dem Fragebogen „Akzeptanz und Zufriedenheit mit der Versorgung durch ein CM aus Perspektive der Angehörigen“, welcher die Ergebnisqualität misst, war das Ziel der vorliegenden Studie. Die in den Interviews genannten Themenkomplexe wurden im Fragebogen ausformuliert und umfassen Aspekte zur Erreichbarkeit des CCM, zu den angebotenen Leistungen und Entlastungsmöglichkeiten als auch Aspekte wie die allgemeine Zufriedenheit und Weiterempfehlungsbereitschaft mit dem CCM. Bisherige quantitative Untersuchungen nutzen Instrumente, die einen indirekten Effekt des CCCM auf die pflegenden Angehörigen messen [17], jedoch wenig Aussagen über die Akzeptanz und Zufriedenheit treffen. Die gewonnenen Ergebnisse sind auf den deutschsprachigen Raum übertragbar. Ein Großteil der befragten Angehörigen war weiblich, und v. a. Ehepartner und eigene Kinder übernehmen in dieser Studie die Unterstützung der zu versorgenden geriatrischen Patienten. Diese Daten zur Geschlechterverteilung als auch der Altersstruktur der hier befragten Angehörigen sind vergleichbar mit den Daten aus der *Studie zur Wirkung des Pflege-Neuausrichtungs-Gesetzes (PNG) und des ersten Pflegestärkungsgesetzes (PSG I)* [18].

Der hier präsentierte Fragebogen zeigt sehr geringe fehlende Werte auf, und die darin enthaltenen Themen weisen eine akzeptable bis sehr gute interne Konsistenz auf und lassen vermuten, dass sie von ihrer Faktorenstruktur her schlüssig sind. Der Fragebogen umfasst zwei wesentliche Komponenten für die Versorgung. Zum einen nimmt er die Auswirkungen, die sich durch Koordination im Rahmen des CM ergeben, in den Blick, und zum anderen thematisiert der Fragebogen die Erreichbarkeit des CCM. Beide Komponenten sind wesentlich, um eine Kontinuität der Versorgung zu gewährleisten, und könnten folglich zu einer Reduktion der wahrgenommenen Belastungen aufseiten der Angehörigen führen. Die Perspektive der Angehörigen spielt in der Versorgungssituation unterstützungsbedürftiger älterer Menschen eine zentrale Rolle [9], sodass es weiterer Forschung bedarf, die neben dem hier vorgestellten Fragebogen Belastungsfaktoren und auch spezifische Aspekte zur Zufriedenheit von Angehörigen miterhebt und mögliche Effekte im Längsschnitt betrachtet.

Bisherige Untersuchungen haben gezeigt, dass ein Hindernis für Angehörige der fehlende Zugang zu adäquater Information und Unterstützung durch Leistungserbringende darstellt [8]. Auch sind die Bedürfnisse von Patienten und deren Angehörigen vielseitig. Es ist zu vermuten, dass CCM in der Lage sein könnten, an diesen Bedürfnissen anzuknüpfen und in Kooperation mit anderen Leistungserbringenden die Qualität der gesundheitlichen Versorgung zu verbessern [20]. Ein CM, welches Angehörige berücksichtigt, eröffnet die Möglichkeit, versorgungsrelevante Informationen individuell näherzubringen. Das heißt, vom Zugang zu therapeutischen Diensten bis hin zur Organisation von Hilfsmittel wird hierbei eine Unterstützung ermöglicht, die zu einer sichtbaren Entlastung der Angehörigen führen kann. Das CM kann möglicherweise dazu beitragen, bestehende parallel existierende Strukturen in dem Sinne aufzuweichen, dass eine gute Strukturierung und Organisation der Versorgung aus der Hand der CCM durch die Vermittlung des Hausarztes angeboten werden. Die Integration der Angehörigenperspektive in die ambulante Versorgungsdynamik kann in Zukunft einen Mehrwert bringen und konsekutiv zu einer verbesserten Versorgungssituation zwischen allen Beteiligten (Leistungserbringende, Patienten, Angehörige) führen.

### Limitationen

Die vorliegende Studie stellt einen ersten Schritt hin zu einem Fragebogen dar, der die Angehörigenperspektive im Rahmen eines CM berücksichtigt bzw. die Akzeptanz und Zufriedenheit der durch das CM angebotenen Unterstützungsleistungen erhebt. Für die Entwicklung des Fragebogens wurden qualitative Interviews durchgeführt, die allerdings nur die Perspektive der CCM sowie der Patienten berücksichtigten. Angehörige wurden mangels Interesse nicht bei den Interviews miteinbezogen. Ein möglicher „confirmation bias“ durch den Interviewer kann nicht ausgeschlossen werden. Die gewonnenen quantitativen Ergebnisse sind unter Vorbehalt eines Selektionsbias zu diskutieren, da in der Regel nur die Angehörigen geantwortet haben, die interessiert und dem Thema gegenüber offen waren und wahrscheinlich von den Angeboten der CCM profitiert haben. Es wurde zudem nicht die Gründe einer Nichtteilnahme erhoben. Es gab kein zusätzliches Erinnerungsschreiben durch die Studienzentrale. Die inhaltliche Überprüfung des konzipierten Fragebogens erfolgte mittels kognitiver Interviews, eine statistische Bestimmung des „content validity index“ wurde nicht vorgenommen. Dem Studiendesign geschuldet, war es nicht möglich, eine Test-Restest-Reliabilität sowie die Änderungssensitivität zu ermitteln. Zukünftige sollten diese Aspekte sowie verschiedenen Aspekte der Validität des Fragebogens in den Fokus nehmen.

## Fazit für die Praxis

Mit dem vorliegenden Fragebogen ist es möglich, die Ergebnisqualität im Rahmen der Umsetzung eines innovativen Versorgungsmodells aus Angehörigenperspektive genauer zu betrachten. Der Fragenbogen zeichnet sich durch seine Kürze aus und umfasst die Akzeptanz und Zufriedenheit der angebotenen Versorgungselemente für Angehörigen in einem Case Management (CM). Durch den Einsatz in anderen Projekten zur Entlastung von Angehörigen durch ein CM könnte die Weiterentwicklung des Fragebogens hinsichtlich relevanter Gütekriterien (Objektivität, Reliabilität und Validität) profitieren.

## Footnotes

1 Kasprick L, Winkelmann C, Häber A, Behrens J. (2018) Musterfachcurriculum Geriatrie – GeriNurse – sektorenübergreifendes Care- und Casemanagement (unveröffentlicht).

## References

[1] Backhaus K, Erichson B, Plinke W (2006). Multivariate Analysemethoden. Eine anwendungsorientierte Einführung.

[2] Bastawrous M (2013). Caregiver burden—a critical discussion. Int J Nurs Stud.

[3] Berthelsen CB, Kristensson J (2015). The content, dissemination and effects of case management interventions for informal caregivers of older adults: a systematic review. Int J Nurs Stud.

[4] Corvol A, Dreier A, Prudhomm J, Thyrian JR, Hoffmann W, Somme D (2017). Consequences of clinical case management for caregivers: a systematic review. Int J Geriatr Psychiatry.

[5] Cronbach LJ (1951). Coefficient alpha and the internal structure of tests. Psychometrika.

[6] Deutsche Gesellschaft für Allgemeinmedizin und Familienmedizin (2018) DEGAM-S3 Leitlinie: Pflegende Angehörige von Erwachsenen. https://www.degam.de/files/Inhalte/Leitlinien-Inhalte/Dokumente/DEGAM-S3-Leitlinien/053-006_Pflegende%20Angehoerige/053-006l_DEGAM%20LL%20Pflegende%20Angeho%CC%88rige_4-3-2019.pdf. Zugegriffen: 10. Dez. 2020

[7] Deutsche Gesellschaft für Care- und Case-Management (2021) Was ist Case Management (CM)? www.dgcc.de/case-management/. Zugegriffen: 22. Jan. 2021

[8] Fjose M, Eilertsen G, Kirkevold M, Grov E (2018). “Non-palliative care”: a qualitative study of older cancer patients’ and their family members’ experiences with the health care system. BMC Health Serv Res.

[9] Häikiö K, Sagbakken M, Rugkasas J (2019). Dementia and patient safety in the community: a qualitative study of family carers’ protective practices and implications for services. BMC Health Serv Res.

[10] Kassenärztliche Bundesvereinigung (2020) Versichertenbefragung 2020. https://www.kbv.de/media/sp/Berichtband_Ergebnisse_KBV_Versichertenbefragung_2020.pdf. Zugegriffen: 10. Dez. 2020

[11] Koro-Ljungberg M, Douglas EP, Therriault D, Malcolm Z, McNeill N (2012). Reconceptualizing and decentering think-aloud methodology in qualitative research. Qual Res.

[12] Kuhlmey A, Dräger D, Winter M, Beikirch E (2010). COMPASS-Versichertenbefragung zu Erwartungen und Wünschen an eine qualitativ gute Pflege. DZA Informationsd Altersfr.

[13] Laag S, Sydow H, Klähn A-K (2019). Transferorientiert fördern: Ein RubiN auf Rezept. GS Gesundh Sozialpolit.

[14] Lukas A, Kilian R, Hay B, Muche R, von Armin CAF, Otto M, Riepe M, Jamour M, Denkinger MD, Nikolaus T (2012). Gesunderhaltung und Entlastung pflegender Angehöriger von Demenzkranken durch ein „initiales Case Management“. Z Gerontol Geriat.

[15] Mayring P (2010). Qualitative Inhaltsanalyse: Grundlagen und Techniken.

[16] Pohontsch N, Meyer T (2015). Das kognitive Interview – Ein Instrument zur Entwicklung und Validierung von Erhebungsinstrumenten. Rehabilitation.

[17] Stojak Z, Jamiolkowski J, Chlabicz S, Marcinowicz L (2019). Levels of satisfaction, workload stress and support amongst informal caregivers of patients receiving or not receiving long-term home nursing care in Poland: a cross-sectional study. Int J Environ Res Public Health.

[18] TNS Infratest Sozialforschung (2017) Abschlussbericht: Studie zur Wirkung des Pflege-Neuausrichtungs-Gesetzes (PNG) und des ersten Pflegestärkungsgesetzes (PSG I). https://www.bundesgesundheitsministerium.de/fileadmin/Dateien/5_Publikationen/Pflege/Berichte/Abschlussbericht_Evaluation_PNG_PSG_I.pdf. Zugegriffen: 10. Dez. 2020

[19] Valentini J, Ruppert D, Magez J, Stegbauer C, Bramesfeld A, Goetz K (2016). Integrated care in German mental health services as benefit for relatives—a qualitative study. BMC Psychiatry.

[20] Wong HH, Yong YH, Shahwan S, Cetty L, Vaingankar J, Hon C, Lee H, Loh C, Abdin E, Subramaniam M (2019). Case management in early psychosis intervention programme: perspectives of clients and caregivers. Early Interv Psychiatry.

